# Covid-19: Fat, Obesity, Inflammation, Ethnicity, and Sex Differences

**DOI:** 10.3390/pathogens9110887

**Published:** 2020-10-26

**Authors:** Indrikis A. Krams, Severi Luoto, Markus J. Rantala, Priit Jõers, Tatjana Krama

**Affiliations:** 1 Latvian Biomedical Research and Study Centre, LV-1067 Riga, Latvia; tatjana.krama@du.lv; 2 Institute of Ecology and Earth Sciences, University of Tartu, EE-51010 Tartu, Estonia; 3 Department of Zoology and Animal Ecology, Faculty of Biology, University of Latvia, LV-1004 Riga, Latvia; 4 School of Psychology, University of Auckland, Auckland 1142, New Zealand; s.luoto@auckland.ac.nz; 5 Department of Biology, Section of Ecology, University of Turku, FI-20014 Turku, Finland; mjranta@utu.fi; 6 Institute of Molecular and Cell Biology, University of Tartu, EE-51010 Tartu, Estonia; priit.joers@ut.ee; 7 Department of Biotechnology, Daugavpils University, LV-5401 Daugavpils, Latvia

**Keywords:** COVID-19, visceral fat, subcutaneous fat, obesity, inflammation, sex differences, ethnicity, mortality, population differences, ACE2

## Abstract

Although obesity is known to be a risk factor for COVID-19 severity, there is an urgent need to distinguish between different kinds of fat—visceral and subcutaneous fat—and their inflammation status in COVID-19. These different fat types have partially diverging biochemical roles in the human body, and they are differentially associated with SARS-CoV-2, which targets the angiotensin-converting enzyme 2 (ACE2) for cell entry. ACE2 is highly expressed in adipose tissue, especially in visceral fat, suggesting an important role for this tissue in determining COVID-19 disease severity. In this perspective article, we discuss group differences in the amount of visceral fat levels and the extent of inflammation in adipocytes of visceral fat tissue, which may, in part, drive population, cross-national, ethnic, and sex differences in COVID-19 disease. It is vital to steer the scientific community’s attention to the effects of visceral fat in creating individual and population differences in COVID-19 severity. This can help researchers unravel the reasons for the reported population, ethnic, and sex differences in COVID-19 severity and mortality.

## 1. Introduction

At the end of 2019, the city of Wuhan in China became the epicenter of the coronavirus pandemic. It appeared initially as an outbreak of atypical pneumonia. Soon after the beginning of the outbreak, Chinese virologists isolated a novel coronavirus (SARS-CoV-2) that causes coronavirus disease (COVID-19). Older age and the presence of several comorbidities are linked with the severity of the disease caused by SARS-CoV-2 [1,2]. Among the most serious comorbid preconditions, the highest mortality rates have been found for diabetes, cardiovascular disease, hypertension, chronic respiratory diseases, cancer, and chronic inflammation [3,4,5]. Importantly, these pathological states are often associated with excess body fat, which causes chronic inflammation in obese people [6,7]. Elevated adiposity is significantly linked with cardiovascular disease, hypertension, diabetes mellitus, and respiratory diseases [8,9]. Moreover, there may be direct biochemical links between hypertension, diabetes, and COVID-19 infection [10]. While a high amount of adipose tissue is a risk factor in some viral diseases such as influenza and hepatitis B [11,12,13], an increase in visceral fat in particular aggravates the COVID-19 infection [3,14,15]. This suggests that the mortality rates of the COVID-19 pandemic [16] may be more highly expressed in countries with high obesity rates [9,16]. Overall and central obesity are risk factors for COVID-19 hospital admission, at least in the United Kingdom [17]. However, this relationship is likely to be more complex, as different types of fat may have different effects on COVID-19 severity in different populations due to their evolutionary history.

## 2. Visceral Fat and Subcutaneous Fat

Although considered for a long time mainly as an energy reservoir, it is clear that adipose tissue has important metabolic and endocrinological roles, with significant differences between different kinds of fat depots. About 90% of body fat is subcutaneous in most people, located just beneath the skin. The remaining fat tissue is called visceral or intra-abdominal fat, and it is located in six visceral fat depots [18]. Accumulation of visceral fat is linked with a variety of diseases [19]. Thus, an important consideration for population and individual health is not necessarily the total amount of fat, but the proportion and perhaps the location of visceral fat. Therefore, it is essential to note that visceral fat is independently linked with worse clinical outcomes upon SARS-CoV-2 infection and is associated with the need for intensive care in patients with COVID-19 [20,21,22].

During the past two decades, the signaling role of adipose tissue has been extensively studied, and one important finding is that visceral fat appears to be endocrinologically more active than subcutaneous fat [23]. Subcutaneous and visceral fat have different evolutionary origins and metabolic functions, producing unique adipocytokine profiles that have separate effects on health [18]. Subcutaneous fat contains a higher concentration of leptin and adiponectin, which act on the brain to suppress appetite and burn stored fat [23]. Visceral fat is generally considered to be more harmful of the two because of its lipotoxicity [24]. It produces higher concentrations of proinflammatory cytokines, which are released into the bloodstream and can lead to auto-amplifying cytokine production (“cytokine storms”) and low-level inflammation, both of which contribute to worse COVID-19 outcomes. Dysfunctional oversecretion of potentially harmful cytokines brought on by impaired metabolism and/or increased visceral fat may constitute a biochemical link between stress and metabolic syndrome [7,24,25]. The reduction in visceral fat is a potential preventive measure for metabolic diseases, which may lower COVID-19 severity due to the strong comorbid association between COVID-19 and metabolic diseases. However, this knowledge alone does not explain the obesity paradox and does not determine the survival prospects of COVID-19 patients with different levels of adiposity [26].

## 3. The Obesity Paradox

Surprisingly, relationships between obesity and mortality in patients with coronary heart disease, heart failure, diabetes, and pneumonia may also be negative—a phenomenon known as the “obesity paradox” [26]. Obese patients usually have high C-reactive protein levels and a high sepsis frequency, suggesting greater inflammation [27]. However, other important details that would prove the negative or positive roles of adipose tissue are generally lacking in studies dealing with the obesity paradox. Therefore, the current understanding might be missing some details to fully comprehend the link between body fat and survival prospects when contracting an infectious disease.

An evolutionarily informed analysis of the origins and functions of different types of fat can provide important information on the role of fat in infectious diseases. According to a novel hypothesis on the evolutionary–developmental regulation of adiposity and body mass, the risk of disease and the need to survive periods of pathogen-induced anorexia (i.e., decreased food consumption, which helps organisms clear pathogens) are the main forces selecting for high adiposity [28]. Evidence on pathogen-induced anorexia indicates that individuals carrying more fat have a better chance of survival when infected, thus providing one explanation for the obesity paradox via improved energy management (namely, stored fat reserves that provide a buffer against periods of pathogen-induced anorexia) [28]. Nevertheless, this hypothesis overlooks the idea that visceral fat may function as an energy depot and fight bacterial and/or viral diseases through its other functions. Although more visceral fat is considered to cause higher local inflammation in the body, its various metabolic functions may bring potentially adaptive benefits in the case of COVID-19 disease. However, this becomes apparent only in between-population comparisons because within-population comparisons show a negative role of visceral obesity in COVID-19 disease [15].

Since greater total fat reserves impair the ability to produce antibodies against a novel antigen [13] and increase susceptibility to disease [18], it appears that adipose tissue has more immunity-related functions than previously considered. First, to explain the obesity paradox, i.e., the better survival of obese individuals under some circumstances [26], we need to consider both the harmful and the beneficial traits of visceral fat and its metabolites, which are capable of providing multi-faceted active protection against parasites and pathogens during disease-borne fasting [8,29]. Although visceral fat metabolism increases general levels of oxidative stress and causes damage to self-tissue, visceral adipose tissue might be an essential part of a life-saving defense mechanism [9,30,31] because this fat depot can help detect and eliminate pathogens and maintain immune homeostasis of the gut microbiome [32,33,34,35].

There is another possible explanation for the obesity paradox associated with population-level differences in SARS-CoV-2 infection. This hypothesis links the inflammation in visceral fat with higher protection against the virus. Adipose tissue might have a dual role in this disease, linked to angiotensin-converting enzyme 2 (ACE2), a transmembrane protein that is used by the SARS-CoV-2 virus to gain entry into cells [36,37]. ACE2 is a cell-surface exoenzyme that converts angiotensin II (Ang II) into vasodilatory angiotensin 1–7 (Ang 1–7). While Ang II exerts proliferative, pro-inflammatory, and pro-fibrotic activities, Ang 1–7 has an opposite function to Ang II within the renin-angiotensin system. In contrast to Ang II, Ang 1–7 provides protective anti-inflammatory and antioxidant effects, and is frequently decreased in metabolic dysfunctions [38]. Ang 1–7 appears to be a crucial component of the molecular mechanisms that fight COVID-19. After viral entry to the cell, ACE2 expression is seriously reduced in lung tissue, pivoting the balance towards circulating angiotensin II that can seriously aggravate lung tissue inflammation. Furthermore, upon SARS-CoV-2 infection, the ongoing cell deterioration attracts macrophages, which often leads to cytokine storms that cause self-damage and inflammation [39]. Lymphopenia and suppression of T lymphocytes further activate macrophages and cause hemophagocytosis and organ failure. Together with a shift towards angiotensin II with all its downstream systemic effects, these processes can be central in determining the severity and lethality of COVID-19 infection. The ability of ACE2 to decrease inflammation is further reduced by the SARS-CoV-2 spike proteins that bind to transmembrane ACE2 [40,41,42].

Adipose tissue in general and visceral adipose tissue in particular have high expression levels of ACE2. We propose that this can affect COVID-19 infection in two ways. First, a high concentration of ACE2 might make visceral adipose tissue potentially harbor immense numbers of virus particles and impact the course of COVID-19 disease in the most adverse way [20,21,22]. On the other hand, being a cell-surface enzyme regulating the AngII/Ang1–7 balance, high levels of ACE2 in visceral fat could function as a reservoir to balance out the negative effects of SARS-CoV-2. Conspicuously, ACE2 amounts are highest in populations with high visceral obesity which also fare best in the COVID-19 pandemic [43,44]. However, it is also the level of activity of ACE2, not just the amount, that is important in its role in COVID-19 infection. ACE2 function is known to be dysregulated in certain metabolic pathologies, mostly due to its removal from cells’ surface via cleavage by the transmembrane disintegrin and metalloproteinase 17 (ADAM17) [42]. Such shedding of ACE2 disrupts its function and is frequently seen in inflammatory states and chronic pathologies associated with increased morbidity in COVID-19 [43,44]. Therefore, the loss of ACE2 function with its dual role in mediating SARS-CoV-2 virus entry and balance of angiotensins might explain the paradoxical connection between adipose tissue and COVID-19 infection when populations with the highest visceral adiposity may have the mildest COVID-19 severity.

Adipose tissue grows by two main mechanisms: hyperplasia (cell number increase) and hypertrophy (cell size increase) [45]. Hypertrophy occurs to meet the need for additional fat storage capacity in the progression of obesity. This type of obesity has long been known to be related to insulin resistance and other aspects of metabolic syndrome, such as inflammation of adipose tissue and an independent predictor for future type 2 diabetes [8,9]. These metabolic alterations might reduce the functional ACE2 pool from cells’ surface [46], preventing SARS-CoV-2 entry to cells and aggravating the systemic effects of COVID-19 infection at the same time [42].

We suggest that the condition and the variation in the level of local inflammation in adipose tissue and especially in visceral adipose tissue [6,7] may be one of the determining factors for the severity of COVID-19. Therefore, we define our hypothesis in the following way: ACE2, a gateway of SARS-CoV-2 virus into the cell, highly expressed in adipose tissue, especially in visceral fat, may make adipose tissue a depot for the virus; however, the accumulation of SARS-CoV-2 in visceral fat depends on whether fat adipocytes have entered an inflammatory state, which reduces the number of transmembrane ACE2 receptors, preventing the infection from invading the adipocytes. On the other hand, metabolically “healthy” adipose tissue with its relatively high expression and undisturbed function of ACE2 might be central in countering the systemic negative effects of COVID-19 infection on, e.g., lung tissue by sustaining the AngII/Ang1–7 balance. This much-discussed dual role of ACE2 in COVID-19 might cause the variability in susceptibility to SARS-CoV-2 in different populations and sexes depending on the amount of their subcutaneous and visceral fat, adipocyte hypertrophy, and the inflammation level of their adipose tissue.

## 4. Ethnicity/Race/Population as a Risk Factor

Human populations differ in the amount of visceral fat normally accumulated in bodies. Visceral adiposity is considerably higher in Southeast Asian populations than in Europe [47]. ACE2 expression is also significantly higher among Asians compared to African Americans and Caucasians [48]. The local climate, pathogen prevalence, and high population density might have favored increased visceral adiposity in Southeast Asian populations as an adaptation against the higher prevalence of food-borne and respiratory infections than in colder climates [49]. Differences in visceral fat, its location, and in liver fat partitioning are also known from other geographic locations and races [50]. As visceral adiposity is directly linked with obesity and cardiometabolic risk profile [50], individuals from different racial and ethnic groups show divergent comorbidities and diverse reactions to infections even if they live in the same area [51]. Substantial differences in COVID-19 infection and mortality rates between Asian and European populations [52] or between white Americans and African Americans [53] have already been shown. African Americans have the lowest visceral adiposity, while it is the highest in Southeast Asia. However, mortality rates are the highest in African Americans and the smallest in Southeast Asian populations [52,53]. While an increase in visceral adipose tissue has been so far uniformly reported as an aggravating factor in COVID-19 [20,21,22], comparisons within populations are uninformative of any possible differences of visceral adipose tissue’s role between populations, and we encourage within-population studies on associations between visceral fat, the inflammation levels in visceral adipose tissue, and COVID-19 severity in more diverse populations, including Southeast Asians.

Obesity-related metabolic abnormalities are caused by fat depots’ location in the body and the adipocyte numbers (hyperplasia) and adipocyte morphology (hypertrophy). It has been shown that adipogenesis is greater in preadipocytes of normal-weight black compared to normal-weight white women but is downregulated to a greater extent in obese black versus obese white women [14,54]. This decline in adipogenic potential has been associated with increased adipocyte hypertrophy and reduced insulin sensitivity in black but not in white women, indicating local inflammation in adipose tissue [55]. Although adipose precursor cells’ recruitment and differentiation in the subcutaneous adipose tissue, rather than merely inflating the cells, would be protective from obesity-associated metabolic complications, adipocytes’ hypertrophic growth usually begins earlier than adipocyte hyperplasia in obesity [42]. Importantly, subcutaneous adipose tissue storage capacity is limited, and further caloric overload leads to excess lipid accumulation in more harmful adipose tissue depots such as visceral, peri-/epicardial, and ectopic sites.

Ethnic and geographic differences in adipose tissue amount and location may arise from African Americans’ ability to store higher amounts of fat in their primary adipose tissue compartment than whites and Southeast Asians. The latter can store the least amounts of subcutaneous fat. These differences in subcutaneous tissue depots’ storage capacity may result from the evolutionary history, previous encounters with coronaviruses [56,57], and ecological conditions of Europeans, Africans, and Southeast Asians. The maximum amount of subcutaneous fat reserves is limited by local inflammation in subcutaneous adipose tissue. This, in turn, is associated with an increase in adipocyte hypertrophy [58], determining the onset of excess fat storing in visceral tissue depots. This means that inflammation in African Americans’ subcutaneous fat is expected at larger subcutaneous adipose tissue reserves than in whites or Southeast Asians. In contrast, visceral fat depots of African Americans are expected to be smaller than in whites and Southeast Asians. Evidence shows Southeast Asians have significantly higher ratios of small-to-larger adipocytes and a higher fraction of very large adipocytes, suggesting local inflammation in their adipose tissue [59], which we hypothesize has some beneficial effect in COVID-19 infection.

## 5. Sex as a Risk Factor

Most complex traits, including disease phenotypes, show some degree of sex differences in humans [56]. So does COVID-19, which is more lethal in males than in females [60]. Visceral fat can partially influence the SARS (in 2003) and the COVID-19 sex differences of higher severity in males compared with females [56,61] because visceral adiposity is significantly higher in males than in females [62]. The greater amount of visceral adipose tissue accumulating in the upper body is a typical visceral fat distribution pattern in men [23]. This fat accumulation pattern may be associated with lower levels of ACE2 in the adipose tissue of men [63,64]. Higher visceral fat loads in the lower body are less associated with metabolic and cardiovascular diseases than higher visceral fat loads in the upper body [65]. Obese men are at a higher risk of developing cardiovascular disease than obese women, even when the amounts of total body fat are equal [66]. Men have higher amounts of circulating ACE2 than women, a sign of dysregulated ACE2 function associated with hypertension and inflammation. However, visceral depots’ storing capacity in most men is rarely exceeded, and their visceral adipocytes may not reach an inflammatory state sufficient to protect against the entry of the SARS-CoV-2 virus. On the other hand, the immune system is sexually dimorphic; women, for instance, have a higher propensity to develop many autoimmune diseases than men [67]. This is often linked with low-grade inflammation making women vulnerable to such diseases as cellulitis, which affects adipose tissue. We suggest that permanent low-grade adipocyte inflammation may decrease ACE2 from the surface of cells through dysregulatory cleavage and make women less susceptible to SARS-CoV-2 than men. Either mechanism can also act complementarily, and more studies are needed to understand why men are more vulnerable to SARS-CoV-2 infection than women [68,69].

As sex is a risk factor for higher severity and mortality in patients with COVID-19, we hypothesize that visceral fat amounts, distribution, and inflammatory state may constitute a biochemical mechanism that influences men’s higher vulnerability to infectious diseases, including COVID-19. Research on the role of subcutaneous and visceral adiposity and fat tissue-related low-grade inflammation in COVID-19 severity must account for sex-related differences in ACE2 expression in the lung and adipose tissue. This important part of the COVID-19 puzzle is still missing [68].

## 6. Conclusions

Medical doctors, virologists, and physiologists should collect data on the severity of COVID-19 in connection with data on the amount and condition of visceral and subcutaneous adiposity of people infected with the SARS-CoV-2 coronavirus [21,22,23]. Current evidence indicates that transmembrane ACE2 and adipose tissue have a paradoxical connection: while ACE2 facilitates COVID-19 disease, making adipose tissue a reservoir of the SARS-CoV-2 virus, the loss of functional ACE2 in the cell membrane, seen in several metabolic dysfunctions, is likely to provide protection against SARS-CoV-2 entry. We emphasize that body mass index (BMI) is an insufficient source of information because BMI cannot distinguish the so-called “bad fat”, visceral fat, from the so-called “good fat”, subcutaneous fat, and their distribution in the body [19,70]. Moreover, studies reporting a positive association between an increase in visceral fat and COVID-19 severity were performed within populations [20,21,22]. Therefore, special attention is needed for between-population variation studies on the role of visceral adiposity in COVID-19 [21,22,23] since significant differences have been reported between African Americans, Southeast Asians, and whites [47,71]. More generally, energy resources and their amount, location, and type are determinants of mortality in pneumonia [72] and most likely also mortality in COVID-19 patients with pneumonia [73] as well as COVID-19 patients’ need for intensive care [21,22,23]. Additionally, high levels of visceral adiposity in Southeast Asia might suggest the beneficial role of visceral fat in fighting COVID-19. Such an increase might be an evolutionary response against previous encounters with coronaviruses in this region [56,57].

As subcutaneous fat is higher in females and visceral fat is higher in males, measuring BMI alone or in conjunction with total body fat percentage will give an inaccurate estimate of sex differences in distributions of specific fat deposits, particularly in visceral fat [62,69]. Due to the cross-national, ethnic, and sex differences in visceral fat distribution, autoimmune diseases, cytokine-related systemic inflammation, and ACE2 expression [30,48,59,69], the approach suggested in this article may help to explain differences in infection and mortality rates across countries, races, ethnicities, and sexes, as well as in defining the evolutionary role of visceral fat in the human body. In the aggregate, adipose tissue heterogeneity needs more attention to enable a complete understanding of the evolutionary roles of specific types of fat reserves; the pathophysiological, maladaptive functions of subcutaneous and visceral adiposity in COVID-19 disease; and the potential beneficial effects of visceral adipose tissue in viral diseases.
